# GWAS Explorer: an open-source tool to explore, visualize, and access GWAS summary statistics in the PLCO Atlas

**DOI:** 10.1038/s41597-022-01921-2

**Published:** 2023-01-12

**Authors:** Mitchell J. Machiela, Wen-Yi Huang, Wendy Wong, Sonja I. Berndt, Joshua Sampson, Jonas De Almeida, Mustapha Abubakar, Jada Hislop, Kai-Ling Chen, Casey Dagnall, Norma Diaz-Mayoral, Mary Ferrell, Michael Furr, Alex Gonzalez, Belynda Hicks, Aubrey K. Hubbard, Amy Hutchinson, Kevin Jiang, Kristine Jones, Jia Liu, Erikka Loftfield, Jennifer Loukissas, Jerome Mabie, Shannon Merkle, Eric Miller, Lori M. Minasian, Ellen Nordgren, Brian Park, Paul Pinsky, Thomas Riley, Lorena Sandoval, Neeraj Saxena, Aurelie Vogt, Jiahui Wang, Craig Williams, Patrick Wright, Meredith Yeager, Bin Zhu, Claire Zhu, Stephen J. Chanock, Montserrat Garcia-Closas, Neal D. Freedman

**Affiliations:** 1 Division of Cancer Epidemiology and Genetics (DCEG), National Cancer Institute (NCI), National Institutes of Health (NIH), Rockville, USA; 2 Essential Software Inc., Center for Biomedical Informatics and Information Technology, NCI, Rockville, USA; 3 Cancer Genomics Research Laboratory, DCEG, NCI, Frederick National Laboratory for Cancer Research (FNLCR), Leidos Biomedical Research, Inc., Rockville, USA; 4 BioProcessing and Trial Logistics Laboratory, FNLCR, Leidos Biomedical Research, Inc. Division of Cancer Prevention, NCI, NIH, Rockville, USA; 5 NCI at Frederick Central Repository, American Type Culture Collection, Rockville, USA; 6 Information Management Services, Inc., Danbury, USA; 7 Division of Cancer Prevention, NCI, NIH, Rockville, USA

**Keywords:** Epidemiology, Cancer genetics, Genetics research, Cancer genetics, Risk factors

## Abstract

The Prostate, Lung, Colorectal and Ovarian (PLCO) Cancer Screening Trial is a prospective cohort study of nearly 155,000 U.S. volunteers aged 55–74 at enrollment in 1993–2001. We developed the PLCO Atlas Project, a large resource for multi-trait genome-wide association studies (GWAS), by genotyping participants with available DNA and genomic consent. Genotyping on high-density arrays and imputation was performed, and GWAS were conducted using a custom semi-automated pipeline. Association summary statistics were generated from a total of 110,562 participants of European, African and Asian ancestry. Application programming interfaces (APIs) and open-source software development kits (SKDs) enable exploring, visualizing and open data access through the PLCO Atlas GWAS Explorer website, promoting Findable, Accessible, Interoperable, and Re-usable (FAIR) principles. Currently the GWAS Explorer hosts association data for 90 traits and >78,000,000 genomic markers, focusing on cancer and cancer-related phenotypes. New traits will be posted as association data becomes available. The PLCO Atlas is a FAIR resource of high-quality genetic and phenotypic data with many potential reuse opportunities for cancer research and genetic epidemiology.

## Background & Summary

From 1993 to 2001, the National Cancer Institute (NCI) Prostate, Lung, Colorectal and Ovarian (PLCO) Cancer Screening Trial enrolled almost 155,000 participants from the U.S. to investigate the impact of cancer screening on cancer-related mortality and secondary endpoints. In addition to these aims, PLCO has been used widely as a prospective cohort. Over the years, PLCO has collected longitudinal health, dietary and lifestyle risk factor data as well as blood, buccal, and pathology samples. These data and samples have been used for studies of cancer etiology and early detection. PLCO has also contributed to many consortia to study the germline genetic architecture of common adult malignancies. As such, numerous previous genotyping efforts have been conducted within the cohort^1–8^.

With the advent of affordable, high-density genotyping platforms, we developed the PLCO Atlas project to enhance the value of PLCO by genotyping all participants who consented to genetic analysis and had available DNA for genotyping. The goal was to build a research resource for studying genetics and gene by environment interactions important for cancer risk. This resource can contribute to the discovery of susceptibility alleles for multiple traits and further define the underlying architecture of genetic susceptibility to these traits. In addition, the resource will be used to evaluate the impact of genetic risk stratification using polygenetic risk scores (PRS) on the screening trial results; and to evaluate the calibration and risk discrimination of cancer risk prediction models that integrate information on genetic and environmental risk factors. As follow-up continues in PLCO, there will be additional cancer diagnoses and deaths occurring with time, yielding larger sample sizes for future research.

## Methods

### The PLCO trial

Study participants were from the NCI PLCO Cancer Screening Trial, a large, randomized trial designed to evaluate if screening for prostate, lung, colorectal, and ovarian cancers lead to mortality reduction for these diseases^9–11^. Almost 155,000 men and women aged between 55 and 74 years were enrolled from 1993 to 2001 at 10 screening centers across the United States (Birmingham, Alabama; Denver, Colorado; Detroit, Michigan; Honolulu, Hawaii; Marshfield, Wisconsin; Minneapolis, Minnesota; Pittsburgh, Pennsylvania; Salt Lake City, Utah; St. Louis, Missouri; Washington, DC). Approximately half of the participants were randomized to the intervention arm and underwent cancer screening, while the other half were in the control arm and received standard medical care. Several self-administered questionnaires were administered at baseline and during follow-up, which collected information on demographics, medical history, family history and various lifestyle and dietary risk factors. Information from these questionnaires have been aggregated and harmonized to produce traits and covariates used in the PLCO Atlas genetic association tests. Blood was collected from screening-arm participants at baseline and at each annual screening visit for up to 5 additional years. In addition, buccal cells were collected from 2000–2003 from control arm participants and again in 2018 from participants in both arms. Cancer incidence and mortality outcomes have been tracked longitudinally with a median follow-up length >18 years for cancer incidence (approximately 44,000 cancers through 2017) and >19 years for deaths (approximately 57,000 deaths through 2018). All cancer diagnoses were confirmed by medical record review and/or via linkage to cancer registries, as previously described^12,13^. All participants provided written informed consent and the study was approved by the Institutional Review Boards at the National Cancer Institute and the 10 screening centers. Additional information about the cohort can be found at https://cdas.cancer.gov/learn/plco/home/.

### GWAS data

**Fig. 1:**
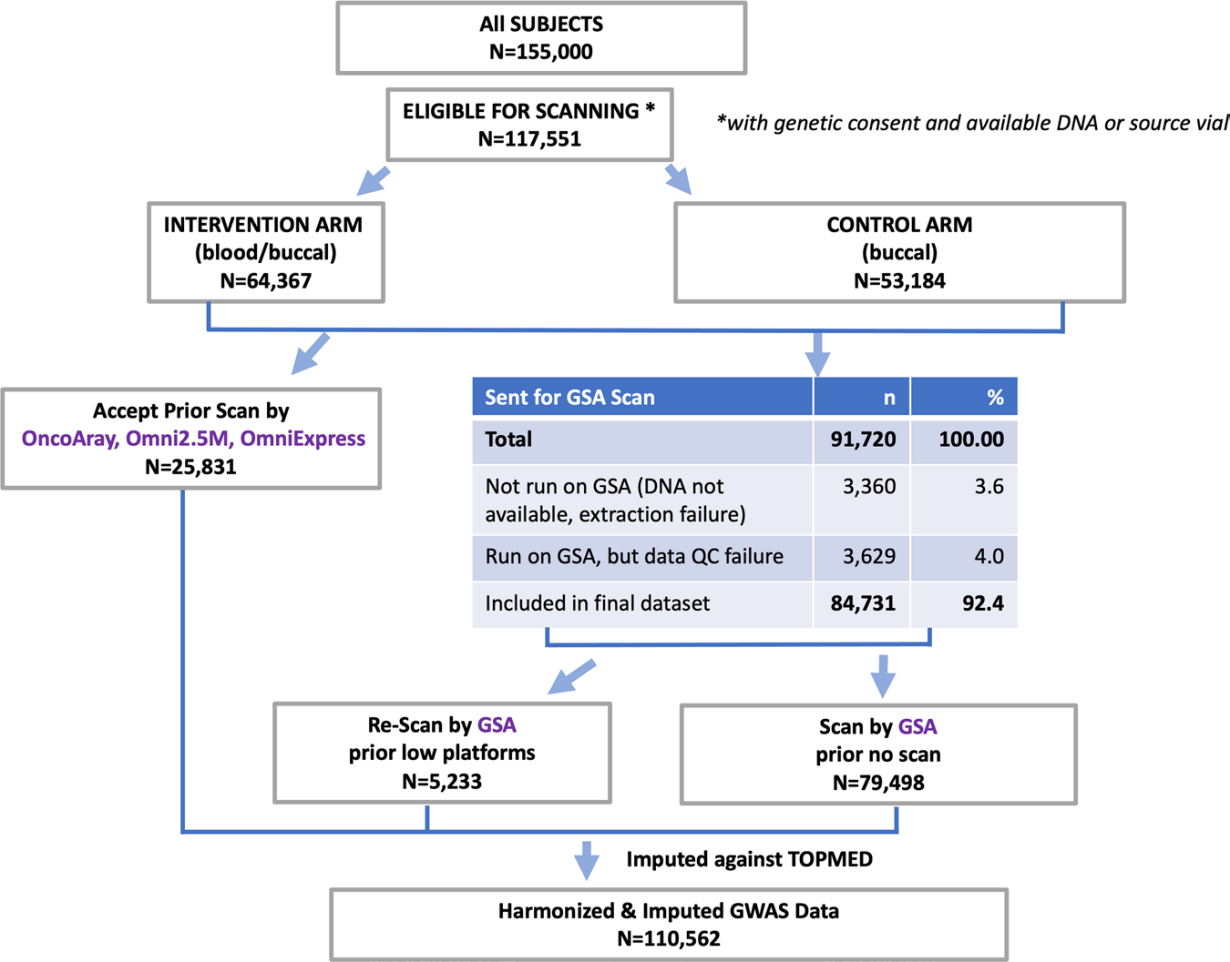
PLCO participants with genotyping data.

The PLCO Atlas genotyping project sought to genotype all PLCO participants with genetic consent and available DNA or source vial (N = 117,551) (Fig. 1). These participants were from the screening arm (N = 64,367) with blood and buccal source material and the control arm (N = 53,184) with only buccal source material. The Atlas project combined genotyping data previously generated by high density arrays for 25,831 participants (OncoArray, Omni2.5 M, and OmniExpress) as part of prior GWAS scans^1–8^ with a new round of genotyping using the Illumina Global Screening Array (GSA) for 84,731 participants who had low-density genotype data (n = 5,233) or no prior genotyping (n = 79,498).

**Fig. 2:**
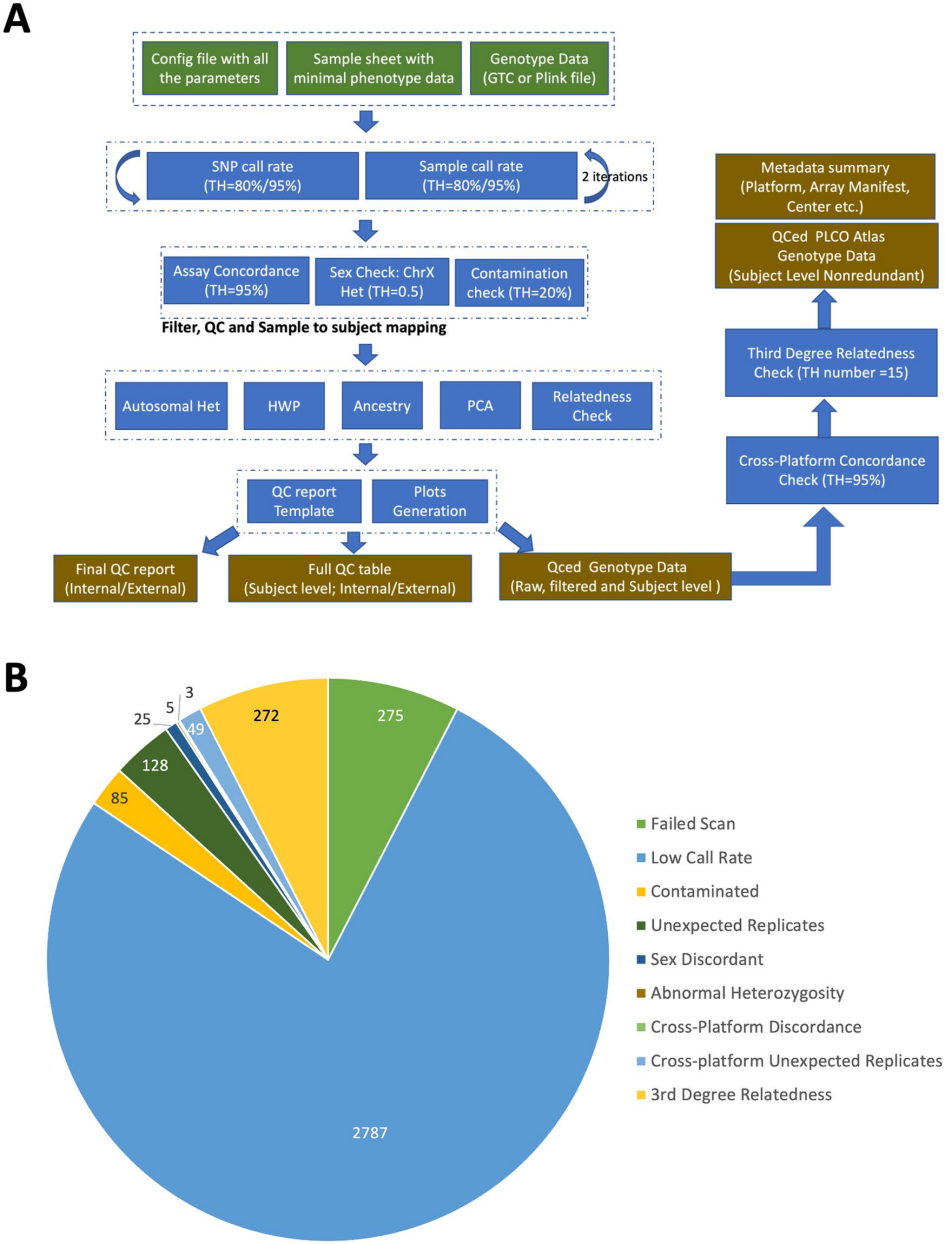
(**a**) PLCO (GSA genotyping platform) quality control workflow. (**b**) PLCO participants excluded due to quality control failures (3,629 participant samples out of 91,720 processed on the GSA platform).

Samples from a total of 91,720 participants were processed for GSA genotyping. DNA extraction was performed using appropriate chemistry based on source material type and automated on the KingFisher Flex Purification System. Extraction protocols were followed using standard operating procedures developed internally in the NCI Division of Cancer Epidemiology and Genetics Cancer Genomics Research (CGR) Laboratory. The predominant DNA sample source was buccal cells (48.4%), followed by buffy coat (39.8%), whole blood (4.2%), and buffy coat and red blood cells (1.4%), as well as previously extracted DNA (2.6%). Of the 91,720 participants whose samples were processed, 3,360 (3.6%) individuals were not genotyped on GSA due to insufficient DNA extracted (N = 2,313) or insufficient material from previously extracted DNA (N = 1,047). In addition, a total of 3,629 (4.0%) individuals were excluded from the final dataset due to quality control failures described below and summarized in Fig. 2a,b, resulting in a total of 84,731 PLCO participants successfully genotyped by GSA.

GSA genotyping was performed at the NCI Division of Cancer Epidemiology and Genetics CGR Laboratory according to Illumina protocols and following internal standard operating procedures. The CGR has extensive experience performing high-throughput Illumina bead-based genotyping having previously genotyped hundreds of thousands of samples. Initial GSA genotyping resulted in an overall failure rate of 1.5% for blood-derived DNA and a failure rate of 13% for buccal-derived DNA. After additional DNA extraction and genotyping to recover sample failures, genotyping was fully completed for a total of 84,731 GSA genotyped individuals.

Extensive quality control filtering was performed for each array to ensure a set of high-quality genotype data for subsequent imputation and association analyses. Detailed quality control steps and the reasons and numbers of exclusions for the GSA platform are described below and summarized in Fig. 2a,b, respectively. For subjects genotyped on GSA, 275 subjects failed to produce valid output files (either .idat and/or .gtc files) during array processing and were excluded from the study. Next, 2,787 subjects were removed after applying a two-stage filter by a completion rate threshold of 0.8 for samples and 0.8 for loci, followed by a further 0.95 filter for samples and 0.95 filter for loci. A sample contamination check was performed using VerifyIDintensity, in which 85 subjects with greater than 20% estimated contamination were removed. Pairwise genotype concordance for all subjects was assessed to identify unexpected replicates, where subjects with a genotype concordance greater than 95% for a set of LD-pruned SNPs were considered replicates. After reviewing concordance check results against the enrolled phenotype data, a total of 128 subjects were removed. Sex was verified by comparing the reported sex with the observed sex based on X chromosome method-of-moments F coefficient from PLINK. The F coefficient is expected to be close to 0.0 for males and 1.0 for females with our threshold set to 0.5 for separating the two populations. Samples that failed the sex concordance check were subject to additional screening for sex chromosome aneuploidies by STR profiling using the AmpFLSTR Identifier assay, resulting in a total of 25 subjects excluded due to sex discordance. Further, a total of 5 subjects were identified to have abnormal heterozygosity by using absolute values from PLINK method-of-moments F coefficients greater than 0.2. Pairwise genotype concordance for all subjects from different datasets within the same platform and across different platforms was also assessed to identify cross-dataset and cross-platform discordant expected duplicates (n = 3) and unexpected replicates (n = 49). Additionally, relatedness was examined using Plink IBS/IBD tests. A total of 272 subjects from the GSA platform were identified with genetic relationships at the pi_hat threshold (0.1) and were removed. Consequently, a total of 3,629 individuals were excluded from the GSA dataset due to quality control failures (Fig. 2b).

Cumulatively, samples from all platforms were filtered to remove abnormal levels of heterozygosity (N = 12), sex discordance (N = 31), within-dataset unexpected duplicates (N = 130), discordant expected duplicates (N = 14), cross-dataset and cross-platform unexpected duplicates (N = 126), and relatedness check (N = 291). A total of 47 subjects with sex-chromosome abnormalities were retained in the dataset for downstream imputation.

After applying QC exclusions to each array, a total of 112,065 DNA samples genotyped across 110,562 unique individuals on a modern, high-density Illumina genotyping array remained (Table 1). For participants genotyped on multiple genotyping arrays (N = 1,192), only genotype data from one array was included in the Atlas project following the prioritization of Global Screening Array (GSA) > OncoArray > Omni2.5 M > OmniExpress (OmniX) to ensure non-redundant subject-level genotyping data. The predominant genotyping array was the GSA (N = 84,731), followed by the OncoArray (N = 16,893), Omni2.5 M (N = 7,211) and OmniX (N = 1,727).

**Table 1:** Distribution of PLCO Atlas participants by ancestral group and genotyping platform.

	GSA	OncoArray	Omni2.5 M	OmniX	Total
African American (Combined)	3,734	774	27	41	4,576
East Asian (Combined)	3,178	297	1	52	3,528
European	75,988	15,669	7,175	1,616	100,448
Hispanic1	189	32	5	3	229
Hispanic2	1,276	94	2	8	1,380
Other	214	22	1	4	241
South Asian	152	5	0	3	160
Total	84,731	16,893	7,211	1,727	110,562

GRAF (https://github.com/ncbi/graf) was used to estimate ancestral group using a set of ancestry informative variants. African American (Combined) includes GRAF outputs of “African” and “African American”. East Asian (Combined) includes GRAF outputs of “East Asian” and “Other Asian or Pacific Islander”. Hispanic1 includes individuals of Dominican or Puerto Rican ancestry whereas Hispanic2 includes individuals of Mexican or Latin American ancestry. GSA = Global Screening Array, OmniX = OmniExpress.

Genetic ancestry for PLCO Atlas participants was determined using GRAF (https://github.com/ncbi/graf) on a set of 10,000 pre-selected fingerprinting variants. GRAF assigned individuals into the following 9 ancestral groups: “African”, “African American”, “East Asian”, “European”, “Hispanic1”, “Hispanic2”, “Other”, “Other Asian”, and “South Asian”. Hispanic1 included individuals of Dominican or Puerto Rican ancestry whereas Hispanic2 included individuals of Mexican or Latin American ancestry. For parsimony and to facilitate downstream analyses, we merged “African” and “African American” into a “African American (Combined)” group and also “East Asian” and “Other Asian” into a “East Asian (Combined)” group. The largest ancestral sets in the PLCO Atlas included European (N = 100,448), African American (Combined) (N = 4,576) and East Asian (Combined) (N = 3,528).

For genotype imputation, we used the TopMed reference panel on the Michigan Imputation Server, which is accessible on the Michigan Imputation Server to all TopMed collaborators. To prepare for genotype imputation on the Michigan Imputation Server (MIS, https://imputationserver.sph.umich.edu), we filtered variants with minor allele frequency ≤ 0.01, variant-level missingness ≥ 0.05, and Hardy Weinberg equilibrium exact test p-value ≤ 1 × 10^−6^ from the imputation input. Data from each genotyping platform were then analyzed using a community-recommended script for aligning data to reference datasets (HRC-1000G-check-bim.pl, from https://www.well.ox.ac.uk/~wrayner/tools/). The script was modified to support TOPMed 5b as a reference panel using a pre-existing test imputation with 1000 Genomes Project subjects versus the TOPMed 5b reference panel. Data were uploaded to the MIS in GRCh37/hg19 and lifted over by the MIS. Pre-phasing using phased reference data from TOPMed release 5b was conducted using EAGLE 2.4. Imputation was conducted against the same reference panel using minimac4 (https://genome.sph.umich.edu/wiki/Minimac4). The “Population” option was set to “EUR” for GSA batches 1–4 that included European ancestry samples, while the option was set to “Other/Mixed” for all other imputations, which consisted of non-European samples or samples of uncharacterized ancestries. The PLCO imputation process took place over several months and was run in different rounds over the span of those months. In total, 110,562 subjects were successfully imputed to the TOPMed 5b reference panel.

Following MIS imputation, raw imputation data were partitioned into subsets according to predicted GRAF genetic ancestry groups to estimate ancestry-specific imputation quality. Ancestry and chip combinations with less than 100 individuals were deemed to have insufficient sample sizes for association testing and removed. After partitioning by ancestry and recomputing imputation quality Rsq values, each platform and ancestry pair was cleaned according to the filtering method described by Kowalski *et al*.^14^. Briefly, all variants with Rsq <0.3 were removed to be consistent with traditional quality filters. Remaining variants were then partitioned into minor allele frequency (MAF) bins (<0.05%, 0.05–0.2%, 0.2–0.5%, 0.5–1%, 1–3%, 3–5%, and >5%) and each bin was filtered, starting at the variant with the lowest Rsq, until the average Rsq of remaining variants within the corresponding MAF bin was at least 0.9. In total, more that 78,000,000 high-quality imputed variants were available for association testing. In addition, we observed high concordance between high quality imputed SNPs from the GSA with genotyped variants present on the OmniExpress arrays, with a median correlation of 1.00 and a mean correlation of 0.984.

Filtered imputed data by platform and ancestry were then converted to bgen format (v1.2) for compatibility with BOLT-LMM and SAIGE for association testing. The resulting final imputed PLCO Atlas Project dataset for association analyses is detailed in Table 2.

**Table 2:** Total resulting imputed PLCO Atlas Project sample size by genotyping array and ancestry group.

Genotyping Array	African American (Combined)	East Asian (Combined)	European	Hispanic1	Hispanic2	South Asian
GSA	3734	3178	75988	102	1276	142
Oncoarray	774	297	15669	NA	NA	NA
OmniX	NA	NA	1635	NA	NA	NA
Omni25	NA	NA	7151	NA	NA	NA

Cells with less than 100 individuals were removed and designated with NA due to challenges fitting association tests with limited sample size.

### Association analysis

Association analyses on the autosomes and X chromosome were carried out using the PLCO pipeline hosted on GitHub (https://github.com/NCI-CGR/plco-analysis). All variants in non-PAR regions of the X chromosome in males were handled by coding these variants as 0/2. Quantitative phenotypes with a sample size of at least 3,000 subjects were analyzed by BOLT-LMM v2.3.4^15^, using linear mixed models on variants with MAF >0.01. The top 20 principal components (generated separately by ancestry) were included as adjustment variables, as well as participant’s age, sex, and study center. Healthy subjects free of any cancer diagnoses throughout the follow-up period were treated as controls for all cancer analyses. Binary and categorical phenotypes were analyzed with SAIGE 0.43.2^16^. We required more than 1,000 subjects and at least 50 cases for each SAIGE phenotype tested. At the variant level, a minimum variant count of 5 and a MAF >0.01 were required for testing. Association analyses were run separately for every GRAF-defined ancestry group, genotyping array, and imputation group. Ancestry-specific results were aggregated by meta-analysis to create overall summary results as well as sex-specific summary result files. Quantile-quantile (Q-Q) plots were generated and lambda values were calculated for each phenotype by linkage disequilibrium score (LDSC) regression^17^.

After association analyses using BOLT-LMM or SAIGE, the SNP column of the GWAS summary files were annotated by a custom tool (https://github.com/NCI-CGR/annotate_rsids_from_linker.git), in the format of rsid:otherAllele:testedAllele (or chr:pos:otherAllele:testedAllele if there was no matching rsid). Population-specific data from the 1000 Genomes Project imputed with the TopMED imputation 5b panel was used to annotate allele frequencies for each tested variant in the GWAS summary statistics using the annotate_frequency program (https://github.com/NCI-CGR/annotate_frequency). Association analyses for every GRAF-defined ancestry group were run separately for each genotyping array and imputation group. While the genotyping arrays and imputation procedures we implemented in the PLCO Atlas captures trait and disease associations with common variants shared across ancestries, associations with population-specific variants and variants with ancestry-specific differences in allele frequencies may not be well captured by this approach.

Currently the PLCO Atlas project hosts association results for 90 diseases and traits, including a comprehensive list of cancer types and subtypes defined by organ site, etiology, and pathology (Table 3). For example, in addition to overall female breast cancer, we’ve included invasive, *in situ*, ductal, lobular, tubular, ER positive, ER negative, PR positive, PR negative, ER positive or PR positive, ER negative and PR negative, HER2 positive, HER2 negative, ER, PR, and HER2 triple-negative, Grade III or Grade IV, Grade II, and Grade I breast cancer. By etiology, we’ve performed GWAS analyses for smoking-related, alcohol-related, obesity-related, height-related, physical activity-related, diabetes-related, and infection-related cancers (overall, and by HPV- or H. pylori-). For smoking-related cancers, for example, we’ve considered cancers of bladder, ureter, kidney, lip, oral cavity, oropharynx, nasopharynx, hypopharynx, larynx, nasal cavity, paranasal sinuses, colorectum, esophagus, gastric, liver (excluding intrahepatic bile duct cancer), lung, myeloid leukemia, ovarian (mucinous), pancreas, and uterine cervix. By pathology, we’ve organized cancers into solid tumors (e.g., carcinomas, sarcomas, or urothelial cancers) and hematologic cancers (e.g., lymphoid or myeloid). Within carcinomas, we further broke down to adenocarcinomas (excluding mixed adenocarcinoma), endocrine or neuroendocrine, and squamous cell cancers.

**Table 3:** Phenotypes and association results available on the PLCO Atlas GWAS Explorer website.

**Cancers by Organ Site**		
All Cancers		
Biliary Cance		
Bladder Cancer		
Breast Cancer	All Female Breast Cancer	
	Female Breast Cancer (Invasive)	
	Female Breast Cancer (Ductal, NOS)	
	Female Breast Cancer (Lobular)	
	Female Breast Cancer (Tubular)	
	Female Breast Cancer (ER Positive: Positive or Equivocal w/positive cells within 1–9% range)	
	Female Breast Cancer (ER Negative)	
	Female Breast Cancer (PR Positive: Positive or Equivocal w/ positive cells within 1–9% range)	
	Female Breast Cancer (PR Negative)	
	Female Breast Cancer (ER Positive or PR Positive)	
	Female Breast Cancer (ER Negative and PR Negative)	
	Female Breast Cancer (HER2 Positive: Positive or Equivocal w/ positive cells within 1–9% range)	
	Female Breast Cancer (HER2 Negative)	
	Female Breast Cancer (ER Negative, PR Negative, HER2 Negative)	
	Female Breast Cancer (Grade III or Grade IV)	
	Female Breast Cancer (Grade II)	
	Female Breast Cancer (Grade I)	
Colorectal Cancer	All Colorectal Cancer	
	Colon Cancer (Distal Colon or Proximal Colon)	
	Colon Cancer (Distal Colon: Descending or Sigmoid)	
	Colon Cancer (Proximal Colon)	
	Rectal Cancer	
Colorectal Adenoma	All Colorectal Adenoma	
	Colorectal Adenoma (Advanced: ≥1 cm or containing high-grade dysplasia or villous, including tubulovillous,elements)	
	Colorectal Adenoma (Non-Advanced)	
Endometrial Cancer	All Endometrial Cancer	
	Endometrial Cancer (Endometrioid Tumors)	
**Glioma Cancer**		
Head and Neck Cancer	All Head and Neck Cancer	
	Cancers of Lip, Oral Cavity, and Pharynx	
	Cancer of Larynx	
Hematologic Malignancies	All Hematologic Malignancies	
	Lymphoid	
	Lymphoid (B-Cell NHL)	
	Lymphoid (CLL)	
	Myeloid	
Kidney Cancer	All Kidney Cancer	
	Kidney Cancer (Renal Cell Carcinoma, NOS)	
**Liver Cancer (excluding Intrahepatic Bile Duct Cancer)**		
Lung Cancer	All Lung Cancer	
	Lung Cancer (Adenocarcinoma)	
	Lung Cancer (Small Cell Carcinoma)	
	Lung Cancer (Squamous Cell Carcinoma)	
	Lung Cancer (Bronchioloalveolar Carcinoma)	
	Lung cancer among never smokers	
	Lung Cancer among former smokers who quit 20 + years ago	
	Lung Cancer among former smokers who quit <20 years ago	
	Lung cancer among current smokers	
Melanoma	All Melanoma	
	Melanoma (Invasive Only)	
Ovarian Cancer	All Ovarian Cancer	
	Ovarian Cancer (High-grade serous: grade III or IV)	
**Pancreatic Cancer**		
Prostate Cancer	All Prostate Cancer	
	Prostate Cancer (Advanced: Gleason 8, 9, 10, or Stage III, IV)	
	Prostate Cancer (Advanced: Gleason 7, 8, 9, 10, or Stage III, IV)	
	Prostate Cancer (Non-Advanced)	
**Thyroid Cancer**		
Upper GI Cancer (Esophageal Cancer or Gastric Cancer)	All Upper GI Cancer (Esophageal Cancer or Gastric Cancer)	
	Esophageal Cancer	
	Esophageal Cancer (Adenocarcinoma)	
	Gastric Cancer	
	Gastric Cancer (Cardia)	
**Cancer by Etiology**		
Alcohol-Related Cancers: Cancers of Lip, Oral Cavity, Pharynx, Larynx, Colorectum, Esophagus (Squamous Cell Carcinoma), Female Breast, Gastric, and Liver (excluding Intrahepatic Bile Duct Cancer)		
Diabetes-Related Cancers: Cancers of Bladder, Colorectum, Endometrium, Female Breast, Liver (excluding Intrahepatic Bile Duct Cancer), and Pancreas		
Height-Related Cancers: Cancers of Colorectum, Endometrium, Female Breast, Kidney, Ovary, Pancreas, Prostate, and Melanoma		
Infection-Related Cancers: EBV, HBV, HCV, H. Pylori, HPV- related cancers		
Helicobacter Pylori-Related Cancer: Gastric Cancer		
HPV-Related Cancers: Cancers of Cervix, Vulva, Vagina, Penis, Anus, Oropharynx, and Tonsil		
Obesity-Related Cancers: Cancers of Lip, Oral Cavity, Pharynx, Larynx, Colorectum, Endometrium, Esophagus (Adenocarcinoma), Female Breast, Gallbladder, Gastric (Cardia), Kidney, Liver (excluding Intrahepatic Bile Duct Cancer), Ovary, Pancreas, Thyroid, Meningioma, and Multiple Myeloma		
Physical Activity-Related Cancers: Cancers of Bladder, Colon, Endometrium, Esophagus (Adenocarcinoma), Female Breast, Gastric (Cardia), Kidney, and Lung		
Smoking-Related Cancers: Cancers of Bladder, Ureter, Kidney, Lip, Oral Cavity, Oropharynx, Nasopharynx, Hypopharynx, Larynx, Nasal Cavity, Paranasal Sinuses, Colorectum, Esophagus, Gastric, Liver (excluding Intrahepatic Bile Duct Cancer), Lung, Myeloid Leukemia, Ovarian (Mucinous), Pancreas, and Uterine Cervix		
**Cancer by Pathology**		
Solid Tumors	All Solid Tumors	
	Carcinomas	All Carcinomas
		Adenocarcinomas (excluding Mixed Adenocarcinoma)
		Endocrine or Neuroendocrine
		Squamous Cell Cancers
	Sarcomas (including Neural Cancers)	
	Urothelial Cancers	
Hematologic Cancers	All Hematologic Cancers	
	Lymphoid	
	Myeloid	
**Selected Lifestyle Factors**		
Body Mass Index at Baseline		
Height at Baseline		
Smoked Cigarettes for ≧ 6 Months (at Baseline)		
Cigarette Smoking Status at Baseline (Never, Former, Current)		
Caffeine from Diet (NDS-R) (mg/day)		
CA-125 Level, First Screen		
PSA Level, First Screen		
Male Hair Pattern at Age 45		

We also include GWAS association results for key cancer risk factors such as baseline status of body mass index, height, cigarette smoking for ≥6 months (never, ever), and cigarette smoking categories (never, former, current), caffeine consumption from diet, and male pattern baldness at age 45, as well as baseline measures of serum PSA level and serum CA-125 level. These initial traits were selected based on available previous data and represent binary, categorical, and continuous traits for the purpose of analytical pipeline development and validity checking. Analyses of additional traits are in progress and association results will be publicly posted as they become available.

### Summary statistics

After association testing and annotation, summary statistic data was imported into a primary MySQL instance using an import script run on the National Institutes of Health (NIH) High Performing Computation Biowulf cluster (https://hpc.nih.gov/) that imported and aggregated participant phenotype metadata and variant association data. Using several parallel processes, each phenotype’s variant association data was aggregated and then indexed. Specific plot views for data visualization, such as the single chromosome summary view in the Manhattan plots and the q-q plots, were generated in this import and indexing process. The results were then pooled into the primary MySQL instance where a snapshot was created in Biowulf using Percona Xtrabackup tool. The snapshot was then uploaded to an Amazon Web Services (AWS) Simple Storage Service (S3) cloud bucket where it was restored to AWS’s Relational Database Service (RDS).

**Fig. 3:**
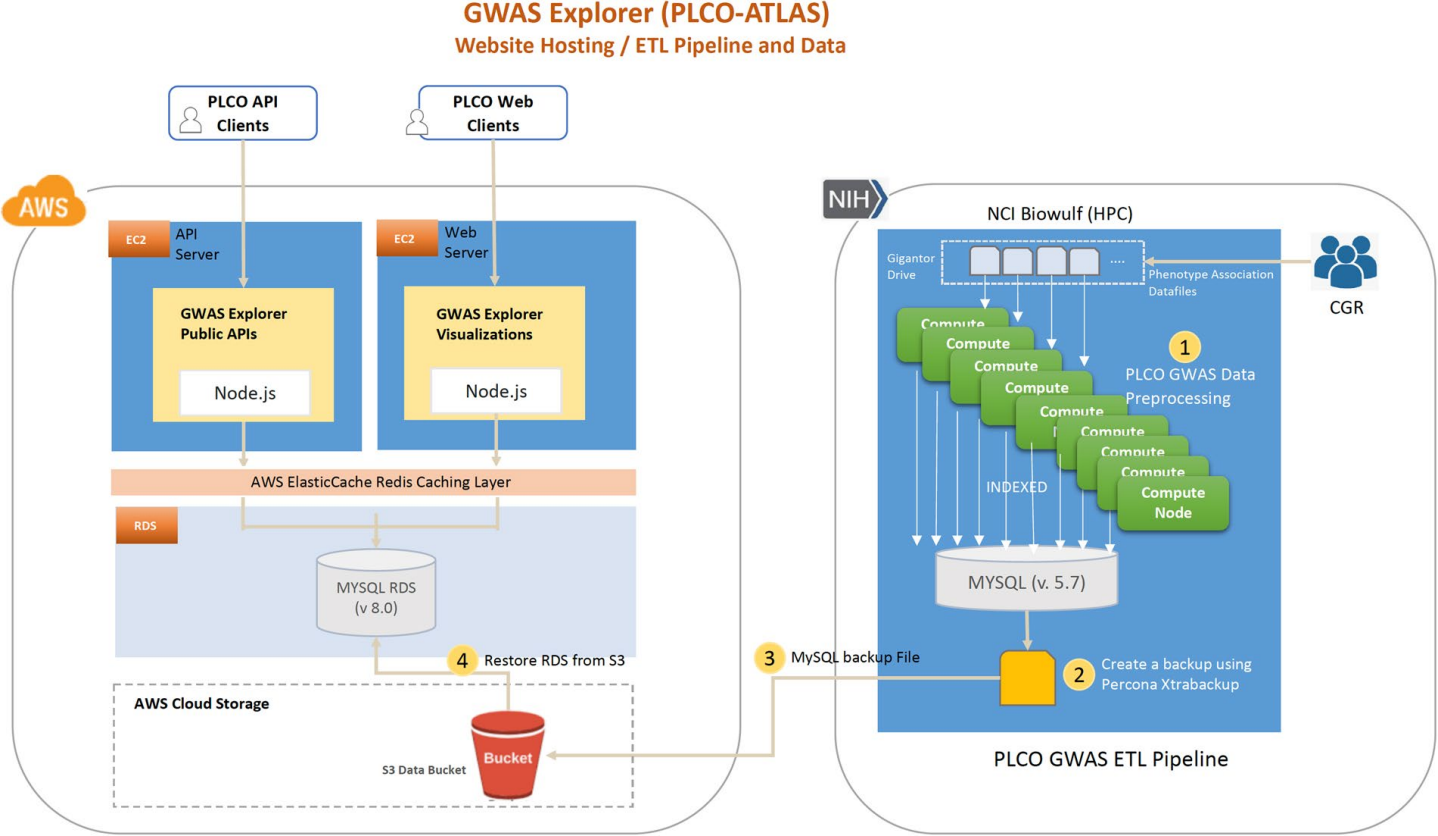
GWAS Explorer data pipeline and website hosting schematic.

All PLCO Atlas summary statistic data is publicly posted on the GWAS Explorer (Fig. 3). The GWAS Explorer is hosted on AWS. It consists of two AWS EC2 servers, an AWS RDS instance, an AWS ElastiCache instance, and an NCI on-premises download server. The website and API are served by each of their own dedicated AWS EC2s. All PLCO data is hosted in a single AWS RDS MySQL instance, which can be scaled-up or duplicated if needed. The GWAS Explorer backend is hosted by Fastify NodeJS, a web application framework like the popular Express framework but optimized for faster API performance. All API routes and database queries defined and utilize MySQL database query logic. Website (internal) requests are routed to a dedicated web server and public API requests are routed to a separate dedicated API server to reduce load on the webserver during periods of high usage. Public API routes are documented with Swagger UI (see **Data Records**). Download requests are routed to a dedicated local NCI download server to reduce egress costs. Additionally, a cache layer is configured using Redis and AWS ElastiCache to reduce server load and speed-up popular requests.

The GWAS Explorer frontend website is built with React NodeJS. All user interface components are developed with Bootstrap. Plots for visualization of participant descriptive characteristics and association data are built in Plotly.js as well as custom solutions; for example, Manhattan plots and gene tracks are built in custom canvas. The quantile-quantile (Q-q), principal component (PC) plots and frequency plots are generated using Plotly.js and the bubble charts in the Browse Phenotypes section are produced using custom D3.js. The Apache service handles and serves all incoming web requests.

## Data Records

All PLCO genotype data is available in dbGaP^18^ under accession number phs001286.v2.p2 (https://identifiers.org/dbgap:phs001286.v2.p2). This public repository allows researchers to apply for access to the individual genotype and imputation data that we used to create the PLCO Atlas. The informed consent document signed by the PLCO study participants allows use of these data by investigators for discovery and hypothesis generation in the investigation of the genetic contributions to cancer and other adult diseases as well as development of novel analytical approaches for GWAS. No IRB review is required for data use. Public posting of genomic summary results is permitted. Companion phenotype data can be requested through the NCI Cancer Data Access System (CDAS) (https://cdas.cancer.gov/plco/). We note that some of the cancer endpoint data used in the PLCO Atlas cannot be shared through CDAS due to restrictions on data use agreements with certain cancer registries. However, summary GWAS statistic data is directly available from the PLCO Atlas GWAS Explorer website (https://exploregwas.cancer.gov/plco-atlas/) as well as accessed directly through API access (https://exploregwas.cancer.gov/plco-atlas/#/api-access).

## Technical Validation

The Illumina genotyping process performed at the CGRL include many systems that aim to ensure reliable, accurate, and high-quality data. Prior to performing the GWAS, dilution and contamination series of control DNA samples were processed through GSA genotyping to establish thresholds for input requirements and level of sensitivity for contamination using VerifyIDintensity. All specimens, from time of arrival at the CGR laboratory through extraction, DNA sample QC, project plating, and genotyping were tracked through Laboratory Information Management Systems (LIMS) specific to CGR and Illumina processes, including sample plate and well locations and array section locations. LIMS uses this information to generate all manifests for downstream analysis automatically, without manual data entry or intervention. Laboratory staff were blinded to sample information as each tube or plate has a unique barcode to identify instance or work to be performed without any identifying information. All laboratory processing, including sample plating, genotype array preparation in plates and on glass arrays, as well as scanning, was performed using automation, which tracks and records sample plate and glass array barcodes for import into LIMS. All processes were calibrated and validated routinely, using controlled methods to standardize processing and reduce the occurrence of human error. Manifests containing all necessary information and metadata to perform analyses were automatically generated from the CGR LIMS for automated QC analysis.

Internal controls were utilized throughout the GWAS, in which at least 1 sample is randomly located per 96-well plate, rotating through a familial trio of subjects and an unrelated subject with DNA extracted from EBV-transformed cell lines. Technical replicates of study subjects were also included, accounting for 2–3% of samples processed, both within and across 96-well plates, also in random well locations. These replicates were located within the 96-well plate to create a unique layout to identify any potential plate mix-ups and distributed across each set of 24 samples run on the GSA chip, so that each chip contained a QC sample. Internal controls and study replicates were utilized during data QC, confirming sex concordance and genotype concordance of internal control and study replicates. For data generated from each 96-well plate to be considered valid, QC samples and study replicates must be >99% concordant with technical replicates.

## Usage Notes

The GWAS Explorer provides three main modules for accessing and interactively viewing the PLCO Atlas GWAS association data, all of which can be accessed from the landing page (https://exploregwas.cancer.gov/plco-atlas/)^19^. All modules were designed for rapid query times, ease of use, and ability to interactively visualize the data.

**Fig. 4:**
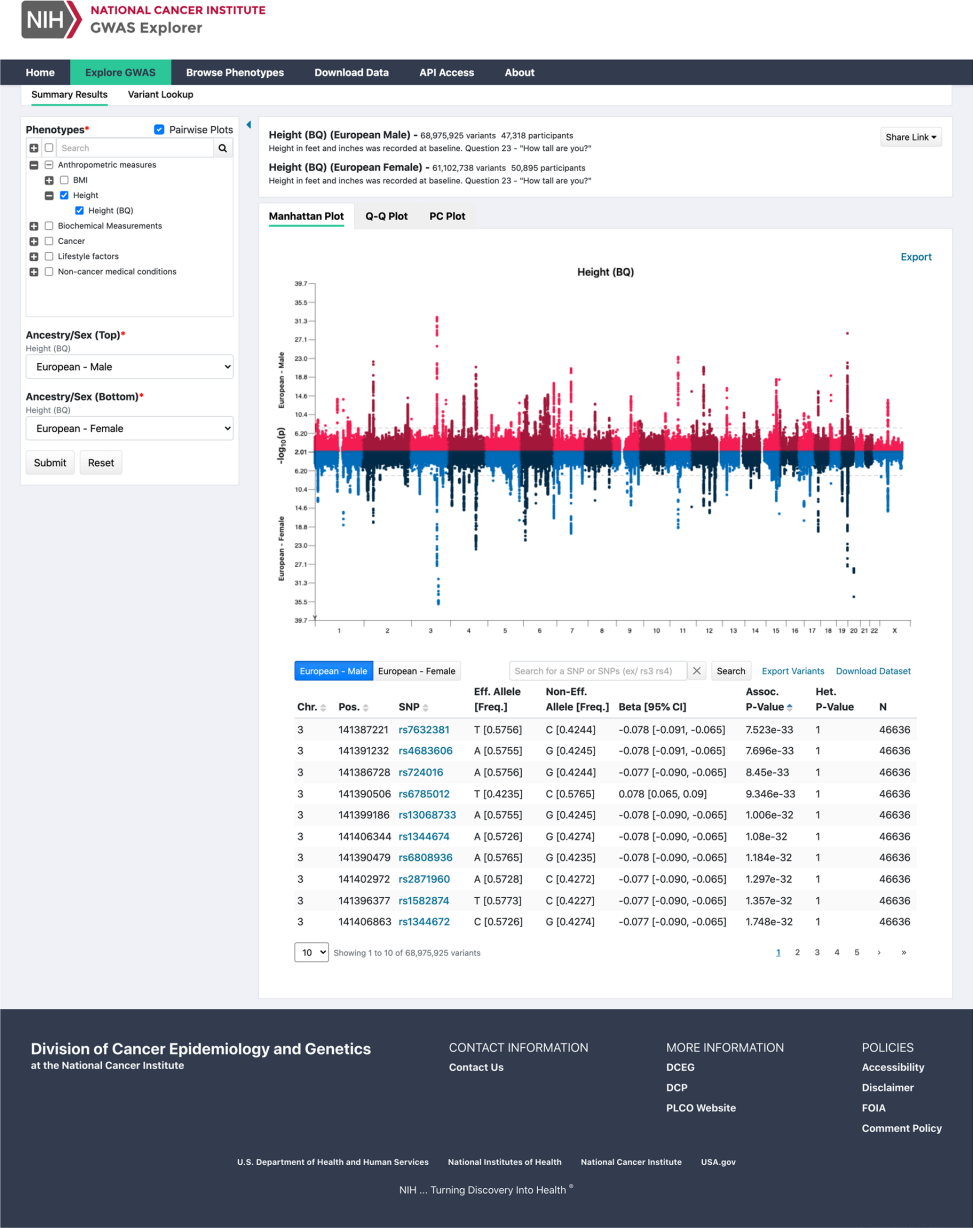
Screenshot of the Explore GWAS results for a pairwise comparison between height in European Males and Height in European females. A Manhattan plot and table of top associations are displayed.

The first module is the “GWAS Results” section where a user navigates a tree menu to select the phenotype, or phenotypes of interest when doing pairwise plots, as well as the desired sex and ancestry group of interest before the Submit button will become active. Once submitted, the query generates a header defining the phenotype and number of participants as well as a series of interactive plots and a table of most significant variants (Fig. 4). The interactive plots include a zoomable Manhattan Plot that displays -log_10_ p-values by chromosomal position along with nearby genes; a quantile-quantile (Q-Q) plot of expected -log_10_ p-values and observed -log_10_ p-values as well as the lambda and number of variants analyzed; and a PC plot that displays all individuals included in the analysis and allows for principal components 1 through 20 to be plotted against each other. Sharable links can be generated to share specific plot views with collaborators. The generated interactive table displays the lowest association p-values for a trait and can be sorted and searched for specific variants. Another tab in the “GWAS Results” section also allows for searching for a specific variant or list of variants by RefSeq (RS) number or chromosomal position (GRCh38).

The second module is the “Browse Phenotypes” section. Here users can get additional information about the participants included in the study by phenotype. A menu tree or bubble plots can be used to browse to a phenotype of interest. Once selected, details and plots for the phenotype will be displayed. Phenotype definition is displayed at the top of the page to clarify how phenotypes were assigned. Plots of overall frequency, frequency by sex and frequency by ancestry are all included. Tables can also be displayed to see the data used to generate the plots. Sharable links can also be generated for the “Browse Phenotype” module.

“Download Data” is the final module and allows users to download the association summary data. Like in the other modules, a tree menu allows users to select a phenotype of interest for download. The submit button begins the download process through the web browser. Up to 5 phenotypes may be selected for download at a time.

In addition to the web interface, the GWAS Explorer also allows for API Access to the data accessible through a REST API. The API enables users to retrieve PLCO Atlas data in their preferred environment and offers more flexibility for querying data than the web interface. The syntax needed to perform API calls is described in the documentation available at https://exploregwas.cancer.gov/plco-atlas/#/api-access. Users can test queries interactively using the web interface before accessing the API programmatically. Many API endpoints require a phenotype_id, which can be obtained by querying the /api/phenotypes route and will return an array of phenotype objects containing the phenotype_id, name, and other properties. Output is returned in JSON format except when specifically indicated.

Further details about the PLCO study and GWAS Atlas are available in the “About” section of the webpage.

## Data Availability

All code for the development and implementation of the GWAS Explorer is available at GitHub in the following repository: https://github.com/CBIIT/nci-webtools-dceg-plco-atlas. All code is in a public repository with no restrictions to access.
